# Class D OXA-48 Carbapenemase in Multidrug-Resistant Enterobacteria, Senegal

**DOI:** 10.3201/eid1701.100224

**Published:** 2011-01

**Authors:** Olivier Moquet, Coralie Bouchiat, Alfred Kinana, Abdoulaye Seck, Omar Arouna, Raymond Bercion, Sebastien Breurec, Benoit Garin

**Affiliations:** Author affiliations: Institut Pasteur, Dakar, Senegal (O. Moquet, C. Bouchiat, A. Kinana, A. Seck, S. Breurec, B. Garin);; Hopital Principal, Dakar (O. Arouna, R. Bercion)

**Keywords:** Nosocomial infections, bacteria, enterobacteria, Senegal, Africa, drug resistance, carbapenemase, OXA-48, letter

**To the Editor:** Class D OXA β-lactamases are characterized as penicillinases that can hydrolyze oxacillin and cloxacillin and are poorly inhibited by clavulanic acid and EDTA. OXA-48 is one of the few members of this family to possess notable carbapenem-hydrolyzing activity (*1*). First described in 2004 in Turkey, OXA-48 has recently started to spread in Europe and the Middle East (*2*). We report the recent emergence of the plasmid-encoded *bla*_OXA-48_ gene in multidrug-resistant *Enterobacteriaceae* recovered from patients in Dakar, Senegal, in hospitals and in the community.

From November 2008 through October 2009, 11 *Enterobacteriaceae* isolates (8 *Klebsiella pneumoniae*, 1 *Escherichia coli*, 1 *Enterobacter cloacae*, and 1 *Enterobacter sakazakii*) with reduced susceptibility to imipenem were identified at the Institut Pasteur (Dakar, Senegal). Antibacterial drug susceptibility was determined by the disk diffusion method and interpreted according to the European Committee on Antimicrobial Susceptibility Testing guidelines (www.eucast.org). Nine isolates were resistant to expanded-spectrum cephalosporins and also to other antibacterial drug classes.

The isolates were recovered from 6 patients with urinary tract infections, 4 patients with surgical infections, and 1 patient with omphalitis. Nine infections were hospital acquired (Le Dantec and Principal Hospitals). Because the patients died before antibacterial drug susceptibility testing could be completed, all 5 patients with surgical infections or omphalitis received only empirical therapy with amoxicillin/clavulanate. One patient with a nosocomial urinary tract infection caused by a co-trimoxazole–susceptible strain was successfully treated with this antibacterial agent. The antibacterial drug regimens of the remaining 4 patients were not known, and they were lost to follow-up. We determined the MICs of imipenem, meropenem, and ertapenem by using the Etest method (AB Biodisk, Solna, Sweden), which showed that 9 isolates were susceptible to imipenem and meropenem but either intermediately susceptible or resistant to ertapenem (Table). The 2 imipenem-nonsusceptible isolates were susceptible or intermediately susceptible to meropenem, and both were resistant to ertapenem.

We used previously described PCRs (*1**,**3**–**7*) to screen for carbapenem-hydrolyzing β-lactamase genes (*bla*_VIM_, *bla*_IMP_, *bla*_KPC_, and *bla*_OXA-48_), as well as plasmid-encoded *bla*_CTX-M_, *bla*_AmpC_, *bla*_OXA-1_, and *bla*_TEM_ β-lactamase genes; the *aac(6′)-Ib* aminoglycoside resistance gene; the quinolone resistance genes *qnrA,B,S*; the tetracycline resistance genes *tetA,B,D*; and class 1 integron. The *bla*_OXA-48_, *bla*_CTX-M_, *bla*_AmpC_, and *aac(6′)-Ib* genes and the variable region of class 1 integron were then characterized by direct DNA sequencing of the PCR products. *bla*_OXA-48_ was present in all 11 isolates. *bla*_VIM_, *bla*_IMP_, and *bla*_KPC_ were not detected. The *qnr* genes were present in 7 isolates resistant to ciprofloxacin. The *aac(6′)-Ib-cr* variant was present in 7 isolates resistant to gentamicin, tobramycin, and ciprofloxacin.

The 9 isolates resistant to expanded-spectrum cephalosporins all harbored the *bla*_CTX-M-15_ gene. The *E. coli* isolate also harbored the plasmid-encoded *bla*_AmpC_ gene ACT-1; *bla*_CTX-M-15_, *bla*_OXA-1_, *bla*_TEM,_ and *aac(6′)lb-cr* were associated in 6 isolates. Long-range PCRs showed that these latter 4 genes were located in the same “multidrug resistance region,” as described in Senegal (*6*). Positive conjugation experiments with sodium azide–resistant *E. coli* J53 showed through PCR results, plasmid DNA extraction, and antibiogram patterns of the obtained transconjugants that *bla*_OXA-48_ was located on a 70-kb self-conjugative plasmid.

The genetic environment of *bla*_OXA-48_ was then investigated by PCR with primers specific for insertion sequence IS*1999* and for the 5′ part of *bla*_OXA-48_ (*1*). *bla*_OXA-48_ was found to be part of a *Tn1999* composite transposon composed of 2 copies of the insertion sequence IS*1999*, as reported (*2*). Further sequencing of the IS*1999* located upstream of *bla*_OXA-48_ showed that it was consistently truncated by the insertion sequence IS*1R*, as initially described in Turkey and more recently in Lebanon and Egypt (*2**,**8*).

*Xba*I pulsed-field gel electrophoresis was then used to study the genetic relatedness of the 8 *K. pneumoniae* isolates. Three isolates had similar restriction profiles and had been recovered from 3 patients concurrently hospitalized at Le Dantec Hospital, suggesting nosocomial transmission. A class 1 integron harboring the *dfrA1* trimethoprim-resistance gene was detected in the 3 clonal isolates.

Together, these findings show the recent emergence of *bla*_OXA-48_ in Senegal in community and hospital settings. They may also suggest the spread of the same major carrying plasmid between the Middle East and Africa. Although 9 of the 11 isolates were found to be susceptible to imipenem on the basis of their MICs, their MICs were nonetheless higher than those of *bla*_OXA-48_–negative isolates. This raises 2 issues. First, these strains might go undetected during routine antibacterial drug susceptibility testing, a problem that could be overcome by using ertapenem, a compound more susceptible to carbapenemases. Second, the clinical efficacy of imipenem on such strains is uncertain. The frequency of acquired carbapenemases, which emerged early after imipenem introduction in Senegal (2008), is probably strongly underestimated, partly owing to the limited availability of reliable clinical laboratories (*9*). Because multidrug resistance is prevalent among *Enterobacteriaceae* isolated in Dakar hospitals (B. Garin, unpub. data) and in rural communities (*6*), the emergence of *bla*_OXA-48_ is a clear cause for concern.

**Table:** Resistance genes and carbapenem MICs of 11 *Enterbacteriaceae* isolates, Senegal, 2008–2009*

Isolate	Species	Origin†	Resistance genes									MIC (µg/mL)		
			*bla*_OXA-48_	*bla*_CTX-M-15_	*bla*_OXA-1_	*bla*_TEM_	*aac-6′-Ib*	*qnr*	*tet*	*dfr*		IPM	MEM	ERT
17176	*Klebsiella pneumoniae*	1	+	+	–	–	–	–	*A,D*	–		1	0.38	1.5
22184	*K. pneumoniae*‡	2	+	+	+	+	*cr*	*S*	*A*	*A1*		1	0.38	1.5
20254	*K. pneumoniae*‡	3	+	+	+	+	*cr*	*S*	*A*	*A1*		1	0.38	1.5
10243	*K .pneumoniae*‡	3	+	+	+	+	*cr*	*S*	*A*	*A1*		1	0.38	1.5
19220	*K. pneumoniae*	2	+	+	+	+	*cr*	*B*	*A,D*	–		0.5	0.19	0.75
18212	*K. pneumoniae*	2	+	–	–	–	–	–	–	–		3	2	12
18220	*K. pneumoniae*	3	+	+	–	+	–	*S*	*D*	–		1	0.38	1.5
06003	*K. pneumoniae*	4	+	–	–	–	–	–	–	–		2	0.25	0.75
HPD	*Enterobacter cloacae*	3	+	+	+	+	*cr*	*B,S*	*B*	–		4	3	8
20247	*Enterobacter sakazakii*	5	+	+	+	+	*cr*	–	–	–		0.5	1	3
24246	*Escherichia coli*	4	+	+	+	–	*cr*	*S*	–	–		2	0.5	2
J53	*E. coli*		–	–	–	–	–	–	–	–		0.12	0.03	0.03
TC	*E. coli*		+	–	–	–	–	–	–	–		0.5	0.19	0.75

*IPM, imipenem; MEM, meropenem; ERT, ertapenem; TC, transconjugants. †Origin: 1, postsurgical visceral infection; 2, postsurgical orthopedic infection; 3, nosocomial urinary tract infection; 4, community-acquired urinary tract infection; 5, omphalocele infection. ‡Clonally related isolates.
